# Reconciling human health with the environment while struggling against the COVID-19 pandemic through improved face mask eco-design

**DOI:** 10.1038/s41598-022-06536-6

**Published:** 2022-02-14

**Authors:** Piergiuseppe Morone, Gülşah Yilan, Enrica Imbert, Leonardo Becchetti

**Affiliations:** 1 Bioeconomy in Transition Research Group – Unitelma Sapienza University of Rome, 00161 Rome, Italy; 2 Department of Chemical Engineering, Marmara University, Göztepe Campus, 34722 İstanbul, Turkey; 3 Department of Economics and Finance, University of Rome Tor Vergata, Via Columbia 2, 00133 Rome, Italy

**Keywords:** Climate-change ecology, Environmental impact

## Abstract

Surgical masks have become critical for protecting human health against the COVID-19 pandemic, even though their environmental burden is a matter of ongoing debate. This study aimed at shedding light on the environmental impacts of single-use (i.e., MD-Type I) versus reusable (i.e., MD-Type IIR) face masks via a comparative life cycle assessment with a cradle-to-grave system boundary. We adopted a two-level analysis using the ReCiPe (H) method, considering both midpoint and endpoint categories. The results showed that reusable face masks created fewer impacts for most midpoint categories. At the endpoint level, reusable face masks were superior to single-use masks, producing scores of 16.16 and 84.20 MPt, respectively. The main environmental impacts of single-use masks were linked to raw material consumption, energy requirements and waste disposal, while the use phase and raw material consumption made the most significant contribution for reusable type. However, our results showed that lower environmental impacts of reusable face masks strongly depend on the use phase since reusable face masks lost their superior performance when the hand wash scenario was tested. Improvement of mask eco-design emerged as another key factor such as using more sustainable raw materials and designing better waste disposal scenarios could significantly lower the environmental impacts.

Shortly after the World Health Organization (WHO) declared COVID-19 a pandemic in March 2020^1^, the production of face masks and, in general, all types of personal protective equipment (PPE) and medical devices (MD) (e.g., gowns, gloves, sanitary stockings) became a global priority. Initially, only health professionals were concerned with using PPE and MD; however, in short order, ordinary citizens dramatically increased their demand for single-use face masks and gloves^2^. In particular, the use of face masks rose drastically, following government mandates for public masking in most countries. As a result, consumption of fossil-based raw materials increased, due to the use of polymeric materials in masks^3^, and a significant amount of waste (including plastic packaging material) was generated, from both healthcare and household units, especially due to the design of most face masks as single-use^4^.

Recently, Benson et al*.*^5^ estimated that approximately 3.4 billion single-use facemasks and face shields are discarded daily across the globe; this figure is also in line with the interpretation of Prata et al*.*^6^, who hypothesized that the pandemic could result in the monthly global consumption and disposal of 129 billion face masks and 65 billion gloves. Moreover, it is worth noting that, despite the introduction of COVID-19 vaccines, the demand for face masks is not expected to decrease, at least in the short term^7^, considering the emergence and spread of new variants^8^. Accordingly, the environmental sustainability of face masks requires careful consideration^9^ to reconcile human health with the environment – a goal that has heretofore engaged both public and private actors^10^. Specifically, the eco-design of face masks must be improved to reduce the environmental impacts linked to their production; at the same time, consumer awareness of their proper use and disposal must be reinforced.

Within the European Union (EU), two main classes of masks are recognized to protect against viral transmission: PPE covered by Regulation EU 2016/425 and MD falling within the scope of the EU legal framework on MD (i.e., Directive 93/42/EEC [MDD], to be replaced by Regulation [EU] 2017/745 [MDR] from 26 May 2021^11^). As shown in Table 1, MD are differentiated into Type I and Type II, according to their bacterial filtration capacity; Type II is further differentiated into Type II and Type IIR, according to whether the mask is splash resistant^12^.

**Table 1:** Specifications of different types of MD.

Test	Type I	Type II	Type IIR
Bacterial filtration efficiency (BFE), (%)	≥ 95	≥ 98	≥ 98
Differential pressure (Pa/cm^2^)	< 40	< 40	< 60
Splash resistance pressure (kPa)	Not required	Not required	≥ 16.0
Microbial cleanliness (cfu/g)	≥ 30	≥ 30	≥ 30

The present study focused on MD, which represents a valuable case for investigation, considering that – as mentioned above—MD are commonly employed by the general public to protect against viral transmission^13^. Specifically, the study quantified the environmental impacts of single-use versus reusable surgical masks to reveal opportunities and limitations along the entire life cycle including the sustainable processing and design of each type. Indeed, reusable masks are not more environmentally sustainable, by definition, than single-use masks, since all life cycle stages should be evaluated in full, i.e., feedstock production, manufacturing, use, and disposal. For this reason, life cycle assessment (LCA)^14,15^ provides a key framework that is commonly applied to assess the environmental sustainability performance of a specific material/product or service^16,17^. In particular, comparative LCA is a way for comparing different options to design products with similar characteristics and functions but with less environmental impacts^18,19^. Moreover, recent contributions to the literature have outlined the importance of LCA for evaluating the environmental implications of face masks^20,21^.

The present study was conducted in Italy, where a significant dispersion of abandoned masks and gloves has been reported, and where the daily use of surgical face masks during the statewide lockdown measures was estimated to reach 40 million pieces per day, alongside 40 million pairs of nitrile or latex single-use gloves^22^. Specifically, considering the yearly consumption of these MD in 2020, a comparative LCA was performed on Type I single-use masks and Type IIR reusable masks, since these two types of masks were described by the interviewed experts as the most widely used in Italy during the time frame considered. Both considered masks (Type I and Type IIR masks) comply with the EN 14,683:2019 standard, and are thereby officially recognized as MD. Single-use (Type I) masks are manufactured using conventional processes, while reusable (Type IIR) masks are produced within the context of an innovative pilot project, called “The Italian Social District” as it integrates economic and productive objectives with the aim of social inclusion, providing work reintegration to several disadvantaged categories, well beyond legal obligations. Specifically, it aims at producing economically viable, environmentally friendly, and socially responsible surgical masks that are effective at protecting against viral transmission. The Italian Social District emerged from the collaboration of 36 firms, and it involved up to 550 workers in its first phase of experimentation and testing. Currently, the project employs 100 manufacturing workers in southern Italy who make and pack the masks, including 46 people in a condition of fragility, participating in a work reintegration program. Since the implementation of the Italian Social District in 2020, the program has generated 7.8 million masks, which have been subsequently approved by the Italian National Institute of Health and used for civil protection.

The present LCA application was based predominantly on primary data (see the “Supplementary Information” and the “Results” section for more details on the data collection), adopting a cradle-to-grave system boundary. Both midpoint (problem-oriented) and endpoint (damage-oriented) impact category scores were evaluated. The main findings showed that reusable face masks demonstrated environmentally superior performance while providing the same level of protection (if not higher) than single-use masks. Yet, it should be noted that the lower environmental impacts of reusable face masks strongly depend on certain consumer behaviors as clearly demonstrated by sensitivity analyses performed.

## Results

### Inventory analysis

Before computing the environmental impacts, we analyzed inventory data and input them into the software program for simulations. With respect to reusable masks, on-site measurements of raw materials, energy requirements for processing (e.g., laying, cutting, sewing, etc.), packaging material configurations, reuse options, cleaning activities and transport distances were provided by the Italian Social District. In particular, requirements for washing the reusable face mask were adapted from Schmutz et al.^9^ in compliance with the information provided by the producer. Moreover, waste disposal scenario data for both types was collected from the preprint by Allison et al.^23^ Finally, inventory data for single-use masks were collected from independent producers via certified laboratories. The final set of background and foreground data are provided in “Supplementary Table S1”.

Single-use face masks consist of three layers of polypropylene non-wovens. The inner and outer fabric layers are Spunbond and the middle layer is 99% filtering Meltblown^24^. Reusable face masks (Type IIR) are also composed of three layers: an internal layer of antibacterial quality cotton, a middle layer of Meltblown, and an external layer of Spunbond. Mask quality is determined by the quality of the component parts and is therefore traceable to the component suppliers. Information on the suppliers and product component types (including certifications and features) is provided in “Supplementary Table S2”. Meltblown (supplied by Ramina) makes up the central part of reusable masks. This component guarantees a filtering performance of more than 99%, which—combined with the high-quality water-repellent anti-drop C6 antibacterial cotton (supplied by Olmetex) of the inner layer—resists up to 10 washes per immersion. These materials, forged together using specialized machinery, enhance Type IIR surgical masks above all others, with respect to their superior performance in the overall trade-off between filtering quality, reusability, and environmental sustainability. Furthermore, the cotton inner fabric of these masks has the same effectiveness as single-use masks in reducing the transmission of respiratory viruses^25^.

Regarding elastic bands, nose clip material (for single-use masks), and fabric layers, no direct datasets are available in the ecoinvent database. Thus, for the present study, non-allergenic latex-free elastic bands, produced using a “polyurethane, flexible foam” process, were assumed. Nose clip material, which is only used for single-use masks, was assumed to be modelled using a “polyvinyl chloride resin (B-PVC)” process. Finally, we assumed that a “polypropylene, granulate” process was used for the TnT Spunbond and Meltblown layers. Regarding packaging materials, reusable face masks are wrapped in biodegradable plastic bags, while single-use masks are packaged in plastic bags. Both types of masks are packaged in sets of 10 and delivered in recycled cardboard boxes. In the present study, packaging materials were introduced to the software as “polyester-complexed starch biopolymer”, “packaging film, low-density polyethylene”, and “corrugated board boxes: 16.6% primary fiber, 83.4% recycled fiber”. For transportation, a “transport, freight, lorry 16–32 metric ton, EURO6” process was assumed from the manufacturing facility and nationwide distribution by road, using Euro 6D vans.

To calculate the number of face masks used in Italy in 2020, we estimated the Italian population at 60.6 million, based on Organisation for Economic Co-operation and Development (OECD) statistics^26^. We assumed one mask per person, per day, for both mask types, according to WHO recommendations^27^. As reusable face masks can be washed up to 10 times without losing their virus filtration performance (according to the manufacturer’s own specification), we assumed the maximum number of washes for the use phase. Accordingly, the total number of face masks used in Italy was calculated at 2.18 and 22.1 billion for reusable and single-use face masks, respectively. The total amount of waste was calculated in terms of the number of used masks, alongside their packaging materials (i.e., plastic wrap and cardboard boxes) (Table 2). Single-use face masks were found to generate almost 10 times more waste for each waste category, relative to reusable face masks.

**Table 2:** Total waste generated from used face masks in Italy, 2020 (kton/year).

	Reusable face masks	Single-use face masks
Masks	15.27	124.64
Recycled cardboard	3.63	33.17
Plastic bags	3.35	25.76
Total	22.25	183.57

With respect to mask use, our basic case scenario was based on WHO recommendations^27^, which stipulate that reusable face masks should be washed daily with soap/detergent and hot (60 °C) water. We assumed that the entire household (2.3 people for Italian case) masks are washed together with other clothes in a standard 7 kg washing machine, following both the literature^9^ and producer instructions. Schmutz et al.^9^ reported that the requirements for a half-full washing machine (a typical situation in Europe) are 84 g detergent, 52.3 L tap water and 1.1 kWh electricity per load. Accordingly, the average washing consumables required for each mask is calculated by normalizing the specified requirements with respect to one mask (i.e., via multiplying a half-full load requirement by 0.2%).

It should be noted, however, that user behavior is not easy to predict and the washing machine might not be always considered as the preferred option. Hence, as a further step, we investigated different user behaviors as sensitivity cases. First of all, hand washing was introduced as the main sensitivity scenario^9,23,28^. In this case, we assumed that the entire household masks will be washed together every day after use, in a bowl of 5 L filled up to 3 L level with water at 60 °C and then rinsed with water without soap/detergent. Approximately 6.24 g of liquid detergent and 6 L of water is required in each manual washing session^23^. Similar to the machine wash case, the average washing consumables required for each mask is calculated by normalizing the specified requirements with respect to one mask (i.e., the requirements per mask per wash are 2.609 L tap water, 2.713 g detergent, 447.7 kJ energy provided by the gas boiler).

Moreover, we also considered other possible user behavior scenarios, assuming that reusable face masks might be washed for more than the recommended lifespan (i.e., 10 washes). Accordingly, a second sensitivity case was modelled for reusable masks washed 15 times prior to disposal. Finally, with reference to single-use masks, we took into consideration a longer period of wearing. Although the recommended face mask use is one mask per day (or 4–8 h), many users wear single-use surgical masks for longer than this recommended period. Thus, in this sensitivity case, we assumed that users would wear the same mask for 2 subsequent days. It should be noted, however, that the latter two sensitivity cases, i.e., concerning longer wearing period of both types, might compromise the protection level of masks and thereby human health.

Regarding the packaging and waste disposal activities, the Italian Social District provided some data from their ongoing studies regarding the biodegradability of packaging materials for reusable (Type IIR) face masks. However, the present study could not consider actual waste disposal activities (i.e., recycling, reuse) due to the lack of approved assessments. Thus, waste disposal was based mainly on previous studies indicating incineration and landfilling as viable options^23,29^. We assumed that contaminated masks and discarded packaging materials would go directly to waste disposal sites, and 43% of mixed waste would be landfilled while 57% of mixed waste would be incinerated^23^. Regarding alternative disposal activities, we considered two sensitivity cases: one that assumed that all masks from each type would be fully incinerated^9,30^ and another that assumed that all masks from each type would be fully landfilled^31^.

## Life cycle impact assessment

The environmental impacts of reusable and single-use face masks were computed using the ReCiPe (H) methodology, in two levels. The following subsections provide a comprehensive discussion of the midpoint (problem-oriented) and endpoint (damage-oriented) impact category scores.

### Comparison of midpoint category scores

The present study considered 18 midpoint category indicators: Climate change, Ozone depletion, Terrestrial acidification, Freshwater eutrophication, Marine eutrophication, Human toxicity, Photochemical oxidant formation, Particulate matter formation, Terrestrial ecotoxicity, Freshwater ecotoxicity, Marine ecotoxicity, Ionizing radiation, Agricultural land occupation, Urban land occupation, Natural land transformation, Water depletion, Metal depletion, and Fossil depletion.

Table 3 presents the overall environmental impacts per functional unit (i.e., the total number of face masks used in 2020). Considering total impacts, single-use face masks performed better than reusable face masks for only 4 impact categories (i.e., Terrestrial ecotoxicity, Agricultural land occupation, Natural land transformation, and Water depletion), while reusable face masks resulted in fewer environmental impacts for the remaining 14 categories. The impact categories for which single-use face masks generated better performance were strictly linked to the use phase requirements and cotton cultivation and processing of reusable face masks.

**Table 3:** Overall environmental impact results.

Impact category	Abbreviation	Unit	Single-use face masks	Reusable face masks
Climate change	CC	kg CO_2_ eq	**9.04E + 08**	*1.47E* + *08*
Ozone depletion	ODP	kg CFC-11 eq	**4.41E + 01**	*1.01E* + *01*
Terrestrial acidification	TAC	kg SO_2_ eq	**2.44E + 06**	*5.20E* + *05*
Freshwater eutrophication	FEU	kg P eq	**8.03E + 04**	*2.14E* + *04*
Marine eutrophication	MEU	kg N eq	**1.75E + 06**	*4.67E* + *05*
Human toxicity	HT	kg 1,4-DB eq	**8.12E + 07**	*2.00E* + *07*
Photochemical oxidant formation	PCOF	kg NMVOC	**2.21E + 06**	*3.50E* + *05*
Particulate matter formation	PMF	kg PM10 eq	**9.09E + 05**	*1.85E* + *05*
Terrestrial ecotoxicity	TECO	kg 1,4-DB eq	*3.90E* + *04*	**1.24E + 06**
Freshwater ecotoxicity	FECO	kg 1,4-DB eq	**8.17E + 06**	*2.03E* + *06*
Marine ecotoxicity	MECO	kg 1,4-DB eq	**7.22E + 06**	*1.62E* + *06*
Ionizing radiation	IR	kBq U235 eq	**5.01E + 07**	*1.07E* + *07*
Agricultural land occupation	ALO	m^2^a	1.64E + 07	**6.02E + 07**
Urban land occupation	ULO	m^2^a	**2.68E + 06**	*6.81E* + *05*
Natural land transformation	NLT	m^2^	*4.95E* + *04*	**1.95E + 05**
Water depletion	WD	m^3^	*7.70E* + *06*	**1.14E + 07**
Metal depletion	MD	kg Fe eq	**1.30E + 07**	*3.83E* + *06*
Fossil depletion	FD	kg oil eq	**3.36E + 08**	*4.02E* + *07*

(Italics indicates the lowest results while Bold indicates the highest results).

**Figure 1:**
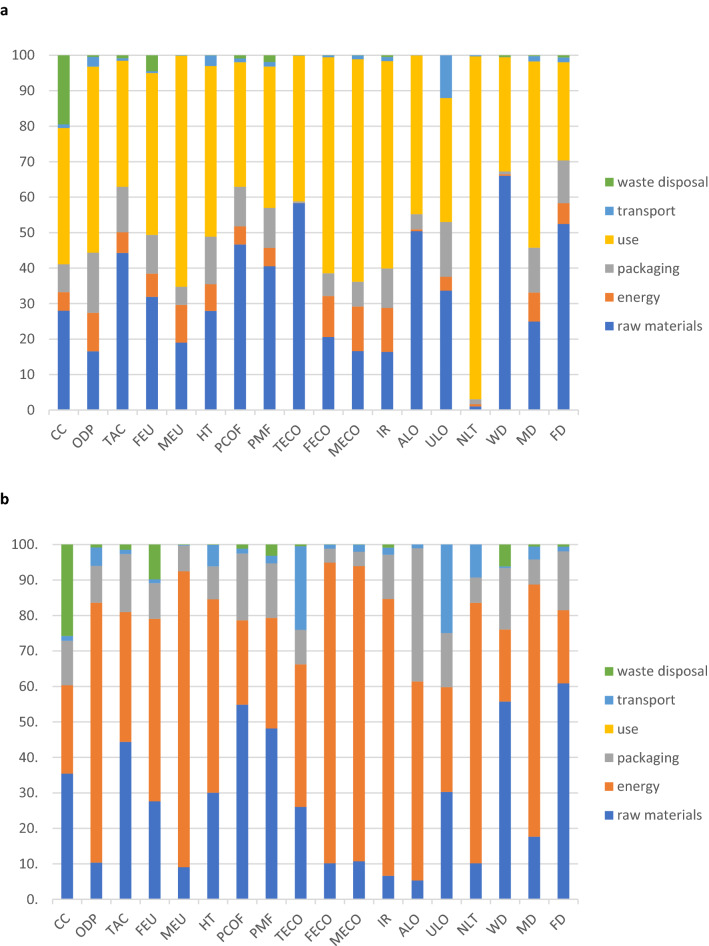
Contribution of each life cycle stage to the midpoint impact category scores. (**a**), Reusable face masks. (**b**), Single-use face masks. (Abbreviations: CC: Climate change, ODP: Ozone depletion, TAC: Terrestrial acidification, FEU: Freshwater eutrophication, MEU: Marine eutrophication, HT: Human toxicity, PCOF: Photochemical oxidant formation, PMF: Particulate matter formation, TECO: Terrestrial ecotoxicity, FECO: Freshwater ecotoxicity, MECO: Marine ecotoxicity, IR: Ionizing radiation, ALO: Agricultural land occupation, ULO: Urban land occupation, NLT: Natural land transformation, WD: Water depletion, MD: Metal depletion, FD: Fossil depletion).

To highlight the effects of each life cycle stage on each midpoint category score, we conducted a contribution analysis for reusable and single-use face masks. The results are presented in Fig. 1 (for further process contribution analyses, please see the figures provided in “Supplementary Fig. S1 and Fig. S2”).

Regarding reusable face masks, the raw materials stage ranks as the second most significant contributor to most of the impact category scores. Especially cotton production and processing activities are responsible for more than half of the overall Water depletion, Terrestrial ecotoxicity, and Agricultural land occupation scores. Concerning single-use face masks, the raw materials and/or energy for production activities were the highest contributors to all impact category scores, and polypropylene and/or polyurethane were the most burden-generating raw materials.

Regarding the manufacturing stage, energy requirements create relatively low burdens compared to other life cycle stages for reusable face masks, while single-use masks were more energy-intensive and created a greater environmental impact on various midpoint categories.

With respect to reusable face masks, the packaging had a relatively low contribution to midpoint scores compared to other life cycle activities. The contribution of packaging on the impact categories mainly pertained to the production of biodegradable plastic bags from starch. Similarly, packaging for single-use face masks had a relatively low contribution to the impact categories, with the exception of Agricultural land occupation. Low-density polyethylene created almost all packaging-related burdens for single-use masks.

The use phase was only considered for reusable face masks, regarding washing activities. For all category scores, it resulted that the use phase alone makes more contribution than one-quarter of the overall impacts. The energy and cleaning agent requirements for washing emerged as the most significant pollutants for the Climate change and Natural land transformation categories, respectively. Therefore, since this phase plays a pivotal role in the decision of shifting from a single-use to reusable face mask, a specific section was devoted to the sensitivity scenarios to include other possible user behavior.

Transportation emerged as a relatively low contributor to almost all categories, for both mask types. For reusable masks, transportation activities contributed to Urban land occupation, and for single-use masks, it contributed to Terrestrial ecotoxicity and Urban land occupation.

Similarly, waste disposal activities made a relatively low contribution to the total midpoint categories for both mask types, except for the categories of Climate change and Freshwater eutrophication. The former category was mainly affected by incineration activities, while the latter was due to landfilling.

#### Comparison of endpoint category results and single scores

The midpoint categories provided a full picture of the environmental impacts associated with all life cycle stages, with a high degree of reliability and certainty. However, the large number of impact categories (i.e., 18) might have increased the degree of complexity in drawing a direct conclusion. Hence, endpoint categories might be used to produce representative scores across different alternatives, with the aim of facilitating decision-making for eco-design^32^. Endpoint categories were evaluated for three areas of protection (i.e., damage categories): human health, ecosystem quality, and resource scarcity. Scores for each area were expressed in disability-adjusted life years (DALYs); local species loss integrated over time (species.yr); and the extra costs involved for future mineral and fossil resource extraction (dollars) for human health, ecosystem quality, and resource scarcity, respectively. A final weighting was applied to produce a representative single score defining the overall environmental load, expressed in million Points (MPt).

Human health scores were calculated as a weighted sum of midpoint indicators concerning increased malnutrition and disease (including respiratory and carcinogenic disease). The indicator scores for reusable and single-use face masks were 268 and 1,559 DALYs, respectively. Ecosystem quality scores were calculated as a weighted sum of midpoint indicators concerning damage to freshwater, terrestrial, and marine species. The indicator scores for reusable and single-use face masks were 2.88 and 7.54 species.yr, respectively. Finally, resource scarcity scores were calculated as a weighted sum of midpoint indicators concerning raw material extraction and energy costs. The indicator scores for reusable and single-use face masks were 6.91 and 56.56 million dollars, respectively.

**Figure 2:**
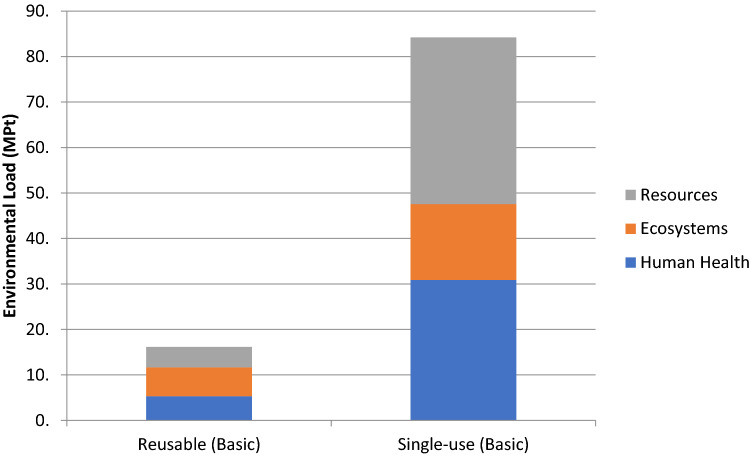
Endpoint level impact assessment results, expressed in a single score. Total environmental load is a weighted score of three damage category scores: human health, ecosystems, and resources.

At the endpoint level, reusable face masks emerged as superior to single-use masks for all three damage categories. The main impacts for the single-use type were linked to the significant consumption of raw materials, energy requirements, and waste disposal activities threatening human health due to a higher amount of waste generation. On the other hand, the contribution of the use phase had a significant impact for reusable masks. The representative single scores for environmental load were 16.16 and 84.20 MPt for reusable and single-use face masks, respectively. In the end, reusable face masks performed better in terms of environmental performance on a single score basis (Fig. 2).

## Sensitivity analyses

**Figure 3:**
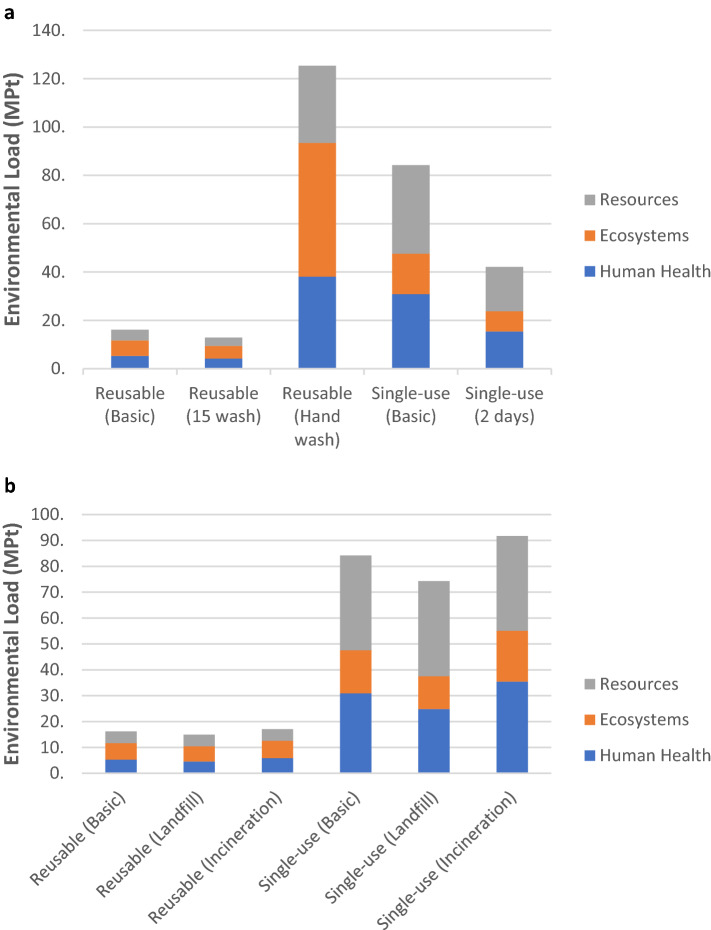
Sensitivity analysis scores. (**a**), User behavior. (**b**), Waste disposal alternatives.

To further elaborate on the results, we performed sensitivity analyses regarding different user behaviors reflecting actual practices and alternative waste disposal scenarios. While only the endpoint category scores (Fig. 3) are discussed here in detail, the results for all sensitivity cases are provided in “Supplementary Tables S3, S4, S5 and S6.”

Regarding user behavior for reusable face masks, environmental impacts significantly increased when the hand wash scenario had been considered. Indeed, the overall environmental impact score for the reusable type was higher compared to the single-use ones, and thereby, in this case, reusable face masks lost their superior performance over the single-use type. An average of nine-fold increase was calculated for each damage category score as well as the total environmental load when compared with the basic scenario due to the extensive use of water, soap, and energy (Table S5). Compared to the single-use masks, the resource scarcity scores were slightly lower while human health scores were slightly higher for reusable ones when washed by hand. The extreme increase was detected in the ecosystem quality scores—three times greater than the single-use scores (Table S6).

As a further step, the total environmental impacts were tested by changing the number of washing before disposing of the reusable mask, i.e., 15 times instead of 10. Consequently, the total impact on each damage category decreased, resulting in a lower overall environmental load shifting from 16.16 to 12.92 MPt, on a single score basis.

For single-use face masks, wearing period directly affected overall environmental impacts, whereby wearing the mask for 2 days lowered all endpoint category scores by half, as expected. However, it should be noted that these alternative behaviors might compromise the protection performance of the masks and hence human health.

Regarding alternative disposal activities, we assumed two sensitivity cases for each mask type: fully incinerated^9,30^ and fully landfilled^31^. This analysis was performed to check whether the mix between landfill and incineration made any significant change to the environmental impacts. For the incineration scenario, scores for the Human health and Ecosystems damage categories were higher than the basic scenario scores for both mask types, while the Resources category score was not affected significantly. In contrast, the landfilling scenario resulted in a lower impact on the Human health and Ecosystems damage categories for both mask types, while the Resources category remained almost unaffected. Hence, on a single score basis, incineration always created a greater environmental load relative to landfilling, with a change range of 10.6 – 14.9% and 5.7 – 17.8% for Human health and Ecosystems damage categories, respectively. Similarly, incineration activities created a 5.7 and 8.9% increase compared to the basic scenario regarding the overall scores for reusable and single-use masks, respectively. The main reason for the higher environmental load was likely to be 14.1 – 18.7% increase in the Climate change category score for reusable and single-use masks, respectively.

## Discussion

The above analysis of midpoint and endpoint impact scores shows that the comparison between single-use and reusable surgical masks, in terms of environmental sustainability, is not trivial. Beyond the higher use of raw materials and the greater consumption of resources needed to produce more pieces per given frequency of use in the case of single-use masks (circularity disadvantage), the two types of masks are also made of different materials and the impact of cleaning reusable masks should be taken into account in a comprehensive way.

Considering these main differences, our findings show that reusable masks outperform single-use masks in terms of overall environmental sustainability since single-use masks perform better on only 4 of the 18 considered midpoint impact criteria: Terrestrial ecotoxicity, Agricultural land occupation, Natural land transformation and Water depletion. However, we observed that the results completely changed in a way that single-use masks performed better in the overall scores when introducing hand washing instead of machine wash (i.e., the basic scenario). This finding is adding saliency to our study by pointing out how the overall environmental impact is highly dependent on washing options. In fact, consumer behavior in cleaning activities has been a matter of great interest not only for medical textiles but also for regular clothing items since the use phase emerged as one of the most important contributors to the overall environmental impact^33^. Prior studies on the LCA of textiles reported that the environmental burdens are significantly affected by the different preferences during the consumer use phase^34^.

Regarding both cleaning options for reusable face masks, the previous studies^9,23^ made various assumptions throughout their calculations, for example, different washing machine characteristics and cleaning material requirements. Notably, a general trend that is in line with our findings clearly emerges as the scores for the reusable type are always worse than the single-use masks when hand wash is selected. Indeed, this result would suggest that the introduction of reusable face masks should be accompanied by an information campaign as well as clear labeling regarding the preferred washing option, to secure a positive overall environmental impact. Similarly, it has been underlined that providing effective consumer guidance on low-impact activities among use phase alternatives is considered a key measure to reduce the total environmental impacts of regular clothes^35^.

Another interesting point is related to the eco-design which could be improved to enhance the environmental performance of reusable masks^31^. For example, Tabatabaei et al*.*^30^ proposed that replacing fossil-based plastics with bio-based plastics may cause a significant decrease in total environmental impacts. Accordingly, polypropylene could be replaced with sustainable cotton fiber^36^. Moreover, improved eco-design regarding waste disposal activities could be also useful to create a tailored scenario for material recycling and/or to produce face masks in a way that facilitates component disassembly and material separation^31^. These findings clearly highlight the importance of eco-design not only considering the activities linked to the raw materials and waste disposal stage but also the use phase, since the masks should always be designed in a way that they can be washed by the machine without losing their protection performance (as in our case study).

Above all, Climate change mitigation is a crucial sustainability domain that demands our focus and represents a critical context for understanding the impact of single-use and reusable masks (in terms of greenhouse gas emissions). As is well known, the ambitious goal of containing the CO_2_ concentration in the atmosphere and limiting the global temperature rise has led the international community to set the goal of net-zero emissions by 2050 (or 2060 for most countries). This implies a tremendous effort to reduce CO_2_ emissions to approximately 54 billion metric tons CO_2_-eq/year at the global level. In part, this goal is still out of reach, since the marginal abatement cost curve is upward sloped (i.e., abatement costs grow significantly as emissions are reduced). It is therefore of utmost importance to analyze and compare the potential benefits (in terms of emission reductions) of single-use versus reusable face masks. Considering the basic scenarios, shifting from single-use to reusable face masks would lead to a yearly emissions reduction of more than 80 percent. This result seems to be in line with the previous studies^28^. Moreover, to increase this percentage, further eco-design practices are needed, such as the application of organic cotton (which would reduce the carbon footprint due to the elimination of pesticides and fossil fertilizers) or recycled cotton components^9^.

Future research should aim at addressing some of the limitations of the present study. First of all, our study tests a specific mask design, for a specific production and waste management scenario in Italy and, thus, it might not be representative of other countries. However, the basic interpretation of our results provided several indications towards improving the environmental performance of surgical masks. Another limitation relates to system boundaries, which were in our case set to include transportation from the manufacturing facility and nationwide distribution. However, transportation of waste to disposal sites and soap/detergent to homes was not considered, due to data deficiencies. Furthermore, consumers’ average use of soap when washing a mask is hard to quantify. Accordingly, in light of the fast-growing number of studies and statistics, a follow-up study should seek to extend the system boundaries to include all transport-related activities. Additionally, with respect to the use phase, public behavior still represents a black box in need of thorough investigation; thus, also general public behavioral studies are required^9^ such as field experiments to directly measure the energy and materials (soap and water) used for cleaning. Moreover, further sensitivity analyses could be performed to consider other important variables, such as different cotton weights.

Finally, above and beyond environmental impacts, other aspects such as face masks’ social impacts and economic costs should be considered to provide a more complete end exhaustive picture of their sustainability. The need for an integrated assessment enabling to cover the three pillars of sustainability has indeed gained momentum in the literature,^37,38^.

Overall, our analysis supports the WHO’s “one health” concept, emphasizing the interdependency between human and environmental health. The findings on the different endpoint categories of single-use versus reusable masks clearly show how a product conceived to improve human health during the COVID-19 pandemic may, in fact, have higher negative indirect consequences on human health if it is conceived with lower environmental standards.

## Methods

The methodological basis of LCA is defined by the standards published by the International Organization for Standardization (ISO). The ISO 14,040 Standard defines LCA as the “compilation and evaluation of the inputs, outputs and potential impacts of a product system throughout its life cycle”^14^. It also highlights the utility of LCA for exploring the possible environmental burdens of a product or service at all stages of the “life cycle”, including resource extraction, material production, use, recycle, and disposal. The basic requirements and guidelines are also introduced by the ISO 14,044 Standard^15^. The present study was carried out in line with the abovementioned ISO 14,040/14,044 guidelines.

According to the ISO 14,040, a typical LCA study is conducted in four successive steps: (i) goal and scope definition, (ii) inventory analysis, (iii) impact assessment, and (iv) interpretation.

## Goal and scope definition

In this first step, the goals and scope of the study are defined, along with the functional unit. The present study aimed at comparing the environmental impacts associated with the use of single-use versus reusable face masks throughout a single year in Italy. The comparison was made within a cradle-to-grave system boundary, which included raw material extraction, transportation (from the manufacturing facility and distribution nationwide), manufacture, use (e.g., cleaning activities, with respect to reusable masks), and waste disposal. The functional unit referred to a quantified description of the primary function of the system under study, represented by the total number of reusable and single-use face masks used by the Italian population in 2020.

## Inventory analysis

Life cycle inventory analysis includes the quantification of: (i) energy carriers and raw materials used; (ii) emissions to the atmosphere, water, and soil; and (iii) different types of land use regarding the functional unit. In the present study, an attributional modelling approach was adopted to examine the environmental impacts generated throughout the life cycle of both types of face masks, and to compare the environmental impacts of these face masks within the same functional unit. During the inventory analysis, two types of data (i.e., foreground and background data) were collected. Foreground data, specific to production activities, was provided by on-site measurements and calculations by the Italian Social District project for reusable face masks. Inventory data for single-use masks were collected from independent producers via certified laboratories. Background data, obtained from the ecoinvent database (v3.01)^39^ and previously published papers, provided further details on generic materials production, energy, transport, and waste management. Inventory processes were input into the SimaPro 8.2.0.0 PhD software as unit processes (i.e., a combination of emissions and resource inputs from the process steps).

## Impact assessment

Life cycle impact assessment (LCIA) interprets and evaluates the degree and significance of the environmental impacts of a product or service. The present study used the ReCiPe impact assessment methodology with a hierarchist (H) perspective V1.12 to calculate environmental impacts, as this reflects the most up-to-date method utilizing both problem-oriented (midpoint) and damage-oriented (endpoint) impact categories^40^. Midpoint category scores have relatively low uncertainty due to their strong relation to environmental flows, while endpoint scores provide potentially better insights into the relevance of environmental flows, albeit with higher uncertainty^32^. In this respect, the two approaches are complementary. The midpoint level indicators chosen for the present study were: Climate change, Ozone depletion, Terrestrial acidification, Freshwater eutrophication, Marine eutrophication, Human toxicity, Photochemical oxidant formation, Particulate matter formation, Terrestrial ecotoxicity, Freshwater ecotoxicity, Marine ecotoxicity, Ionizing radiation, Agricultural land occupation, Urban land occupation, Natural land transformation, Water depletion, Metal depletion, and Fossil depletion. These midpoint level indicators were then combined into three endpoint damage categories: human health, ecosystems, and resource surplus costs. Finally, the three endpoint categories were normalized, weighted, and aggregated into a single score representing the overall system for comparing single-use versus reusable face masks. The SimaPro 8.2.0.0 PhD software package was used to estimate the midpoint and endpoint level environmental impacts, per functional unit.

## Interpretation

The final phase of LCA evaluates the results of either an inventory analysis or an impact assessment, in the context of the study goals. It also aims at proposing recommendations and preparing the final reporting of the results. A detailed discussion of the interpretation phase of the present study is provided in the “Results” section.

## Supplementary Information

Supplementary Information.

## Data Availability

All data collected, generated, and analyzed during this study are included in the “Supplementary Information”. Additional questions about the data may be directed to the corresponding author.
