# Supplementary material for: Reconciling human health with the environment while struggling against the COVID-19 pandemic through improved face mask eco-design
Source: Sci Rep. 2022 Feb 14;12:2445. doi: 10.1038/s41598-022-06536-6 (PMC8844361; doi:10.1038/s41598-022-06536-6)
Supplement: Supplementary file 1 — Supplementary Information. [file 41598_2022_6536_MOESM1_ESM.pdf]

Supplementary Information

Reconciling human health with the environment while struggling against the COVID-19 pandemic through improved face mask eco-design

Piergiuseppe Morone[1,*], *Gülşah Yilan*[1,2], *Enrica Imbert*[1], *Leonardo Becchetti*[3]

[1]Bioeconomy in Transition Research Group – Unitelma Sapienza University of Rome, 00161 Rome, Italy

[2]Department of Chemical Engineering, Marmara University, Göztepe Campus, 34722 İstanbul, Turkey

[3]Department of Economics and Finance, University of Rome Tor Vergata, Via Columbia 2, 00133 Rome, Italy

*Corresponding author. E-mail: piergiuseppe.morone@unitelmasapienza.it

Table S1. The inventory data for the face masks considered.

	Model 1		Model 2	
**Specification**	**Reusable face mask (Type IIR)**		**Single-use face mask**	
**Raw materials**	**Material**	**Weight, g**	**Material**	**Weight, g**
Layer 1	Cotton[i]	2	TnT Spunbond (Polypropylene)[ii]	1.5
Layer 2	Meltblown (Polypropylene)[iii]	2	Meltblown (Polypropylene)[iii]	1.5
Layer 3	TnT Spunbond (Polypropylene)[ii]	2	TnT Spunbond (Polypropylene)[ii]	1.5
Nose clip material	None	0	Plastic (PVC) [iv]	0.14
Laces or elastics for the ears	Elastan latex free (Polyurethane)[v]	1	Elastan latex free (Polyurethane)[v]	1
Total		7		5.64
**Energy**	**Amount**		**Amount**	
Electricity consumption[vi]	5.54	kWh/1000 mask	16.15	kWh/1000 mask
**Packaging**	**Material**	**Weight, g**	**Material**	**Weight, g**
Box	Recycled cardboard[vii]	2.5	Recycled cardboard[vii]	1.5
Wrap	Biodegradable plastic bags[viii]	0.0154	PPE envelopes[ix]	0.0116
**Transport**	**Mode**	**Distance (km)**	**Mode**	**Distance (km)**
	Truck[x]	600[xi]	Truck[x]	600[xi]
**Use**				
Reuse	10 times[xii]			
		Per mask per wash		
Washing	Soap[xiii]	0.168 g[xiv]		
	Water[xv]	0.1046 L[xiv]		
	Energy[vi]	0.0022 kWh[xiv]		
**Waste**	**Component**	**Weight, kton**	**Component**	**Weight, kton**
	Masks	15.27	Masks	124.64
	Recycled cardboard	3.63	Recycled cardboard	33.17
	Biodegradable plastic bags	3.35	PPE envelopes	25.76
**Disposal**				
Landfill[xvi]	43%[xvii]		43% [xvii]	
Incineration[xviii]	57%[xvii]		57% [xvii]	

[i] Cotton fibre {GLO}| market for | Alloc Def, U
[ii] Polypropylene, granulate {GLO}| market for | Alloc Def, U
[iii] Polypropylene, granulate {GLO}| market for | Alloc Def, U
[iv] Polyvinylchloride resin (B-PVC), bulk polymerisation, production mix, at plant RER
[v] Polyurethane, flexible foam {GLO}| market for | Alloc Def, U
[vi] Electricity, low voltage {IT}| market for | Alloc Def, U
[vii] Corrugated board boxes, technology mix, prod. mix, 16,6 % primary fibre, 83,4 % recycled fibre EU-25 S
[viii] Polyester-complexed starch biopolymer {GLO}| market for | Alloc Def, U
[ix] Packaging film, low-density polyethylene {GLO}| market for | Alloc Def, U
[x] Transport, freight, lorry 16-32 metric ton, EURO6 {GLO}| market for | Alloc Def, U
[xi] Estimated average distance including nationwide distribution and the transportation from manufacturing facility to terminal
[xii] Provided in the technical sheet by the manufacturer. It is available from the authors upon request.
[xiii] Soap {GLO}| market for | Alloc Def, U
[xiv] Based on the calculations of Schmutz et al (2020)
[xv] Tap water {Europe without Switzerland}| market for | Alloc Def, U
[xvi] Landfill of plastic waste EU-27
[xvii] Based on the calculations of Allison et al. (2020)
[xviii] Waste incineration of plastics (PE, PP, PS, PB), EU-27
Unmarked data consists of primary data, i.e., provided by the manufacturer, and/or certified laboratories, and/or own calculations.

Table S2. Information on the suppliers and product component types.

Supplier	Component	Certification and Features
ALPHAPACK	TNT Spunbond	ISO 10993-5:2009 - EN 14683: 2019 - EN ISO 11737-1:2018 - UNI EN ISO 10993-1:2010 - UNI EN ISO 10993-10:2013 - UNI EN ISO 10993-12:2012
OLMETEX	Cotton	UNI EN ISO 3758 – ANTI-DROP C6 ANTIBACTERIAL
RAMINA s.rl.	TNT Meltblown	NWSP 130.1 (15) - ANT ISO 9073-3 - NWSP 070.1 R0(15) - ASTM D5729-97(2004)e1
SOFT CHEMICALS S.r.l	Softgard TZO: Fluorinated Polymers Excluding PFOA and PFOS	ISO 9001 2015 - UNI EN ISO 9001: 2015
AGENCY s.r.l.	Flat White 4mm Elastic	OEKO - REACH
AL PACK s.r.l	Pack	Recycled
STEROX	Sterilization	UNI EN 1422, UNI EN ISO 11135-1, UNI EN ISO 10993-7

Table S3. Impact assessment results for reusable face masks, sensitivity cases and relative differences in the scores (ReCiPe (H) Midpoint method)

Impact category	Unit	Basic	15 Wash		Hand wash		Incineration		Landfill	
Climate change	kg CO_2 eq	1.47E+08	1.17E+08	-20.5%	1.19E+09	709%	1.67E+08	14.1%	1.19E+08	-18.7%
Ozone depletion	kg CFC-11 eq	1.01E+01	8.49E+00	-15.9%	2.26E+01	124%	1.01E+01	0.0%	1.01E+01	0.0%
Terrestrial acidification	kg SO_2 eq	5.20E+05	4.08E+05	-21.5%	1.77E+06	241%	5.21E+05	0.1%	5.19E+05	-0.2%
Freshwater eutrophication	kg P eq	2.14E+04	1.75E+04	-18.1%	8.63E+04	304%	2.04E+04	-4.5%	2.26E+04	6.0%
Marine eutrophication	kg N eq	4.67E+05	4.13E+05	-11.6%	1.96E+06	319%	4.67E+05	0.0%	4.67E+05	0.0%
Human toxicity	kg 1,4-DB eq	2.00E+07	1.66E+07	-17.3%	7.72E+07	285%	2.00E+07	0.0%	2.00E+07	0.0%
Photochemical oxidant formation	kg NMVOC	3.50E+05	2.74E+05	-21.6%	1.47E+06	320%	3.50E+05	0.0%	3.50E+05	-0.1%
Particulate matter formation	kg PM10 eq	1.85E+05	1.48E+05	-20.0%	8.08E+05	336%	1.83E+05	-1.3%	1.88E+05	1.7%
Terrestrial ecotoxicity	kg 1,4-DB eq	1.24E+06	9.99E+05	-19.6%	8.94E+06	620%	1.24E+06	0.0%	1.24E+06	0.0%
Freshwater ecotoxicity	kg 1,4-DB eq	2.03E+06	1.77E+06	-13.0%	6.62E+06	226%	2.03E+06	0.0%	2.03E+06	0.0%
Marine ecotoxicity	kg 1,4-DB eq	1.62E+06	1.42E+06	-12.4%	4.73E+06	192%	1.62E+06	0.0%	1.62E+06	0.0%
Ionising radiation	kBq U235 eq	1.07E+07	9.20E+06	-13.9%	2.55E+07	139%	1.07E+07	0.0%	1.07E+07	0.0%
Agricultural land occupation	m^2a	6.02E+07	4.92E+07	-18.4%	4.49E+08	646%	6.02E+07	0.0%	6.02E+07	0.0%
Urban land occupation	m^2a	6.81E+05	5.33E+05	-21.7%	2.65E+06	289%	6.81E+05	0.0%	6.81E+05	0.0%
Natural land transformation	m^2	1.95E+05	1.93E+05	-1.1%	2.98E+06	1424%	1.95E+05	0.0%	1.95E+05	0.0%
Water depletion	m^3	1.14E+07	8.84E+06	-22.6%	8.38E+07	634%	1.15E+07	0.4%	1.14E+07	-0.5%
Metal depletion	kg Fe eq	3.83E+06	3.22E+06	-15.8%	2.16E+07	465%	3.82E+06	-0.2%	3.84E+06	0.3%
Fossil depletion	kg oil eq	4.02E+07	3.05E+07	-24.1%	2.88E+08	618%	4.01E+07	-0.2%	4.03E+07	0.2%

Table S4. Impact assessment results for single-use face masks, sensitivity cases and relative differences in the scores (ReCiPe (H) Midpoint method)

Impact category	Unit	Basic	2 Days		Incineration		Landfill	
Climate change	kg CO_2 eq	9.04E+08	4.52E+08	-50%	1.07E+09	18.7%	6.80E+08	-24.8%
Ozone depletion	kg CFC-11 eq	4.41E+01	2.20E+01	-50%	4.41E+01	0.0%	4.41E+01	0.0%
Terrestrial acidification	kg SO_2 eq	2.44E+06	1.22E+06	-50%	2.45E+06	0.3%	2.43E+06	-0.3%
Freshwater eutrophication	kg P eq	8.03E+04	4.01E+04	-50%	7.24E+04	-9.8%	9.06E+04	12.9%
Marine eutrophication	kg N eq	1.75E+06	8.77E+05	-50%	1.75E+06	0.0%	1.75E+06	0.0%
Human toxicity	kg 1,4-DB eq	8.12E+07	4.06E+07	-50%	8.11E+07	0.0%	8.12E+07	0.0%
Photochemical oxidant formation	kg NMVOC	2.21E+06	1.11E+06	-50%	2.21E+06	0.1%	2.21E+06	-0.1%
Particulate matter formation	kg PM10 eq	9.09E+05	4.54E+05	-50%	8.89E+05	-2.1%	9.34E+05	2.8%
Terrestrial ecotoxicity	kg 1,4-DB eq	3.90E+04	1.95E+04	-50%	3.89E+04	-0.4%	3.92E+04	0.5%
Freshwater ecotoxicity	kg 1,4-DB eq	8.17E+06	4.09E+06	-50%	8.17E+06	0.0%	8.17E+06	0.0%
Marine ecotoxicity	kg 1,4-DB eq	7.22E+06	3.61E+06	-50%	7.22E+06	0.0%	7.22E+06	0.0%
Ionising radiation	kBq U235 eq	5.01E+07	2.51E+07	-50%	5.01E+07	0.0%	5.01E+07	0.0%
Agricultural land occupation	m^2a	1.64E+07	8.19E+06	-50%	1.64E+07	0.0%	1.64E+07	0.0%
Urban land occupation	m^2a	2.68E+06	1.34E+06	-50%	2.68E+06	0.0%	2.68E+06	0.0%
Natural land transformation	m^2	4.95E+04	2.48E+04	-50%	4.95E+04	0.0%	4.95E+04	0.0%
Water depletion	m^3	7.70E+06	3.85E+06	-50%	8.05E+06	4.5%	7.24E+06	-6.0%
Metal depletion	kg Fe eq	1.30E+07	6.50E+06	-50%	1.29E+07	-0.5%	1.31E+07	0.7%
Fossil depletion	kg oil eq	3.36E+08	1.68E+08	-50%	3.36E+08	-0.2%	3.37E+08	0.2%

Table S5. Impact assessment results for reusable face masks, sensitivity cases and relative differences in the scores (ReCiPe (H) Endpoint method)

**Damage category**	**Unit**	**Basic**	**15 Wash**		**Hand wash**		**Incineration**		**Landfill**	
Human Health	DALY	268	213	-20.3%	1926	803%	296	10.6%	230	-14.1%
Ecosystems	species.yr	2.88	2.39	-17.3%	25.00	947%	3.05	5.7%	2.67	-7.5%
Resources	M$	6.91	5.27	-23.8%	49.28	835%	6.90	-0.2%	6.93	0.2%
**Total**	MPt	16.16	12.92	-20.1%	125.36	870%	17.08	5.7%	14.94	-7.5%

Table S6. Impact assessment results for single-use face masks, sensitivity cases and relative differences in the scores (ReCiPe (H) Endpoint method)

**Damage category**	**Unit**	**Basic**	**2 Days**		**Incineration**		**Landfill**	
Human Health	DALY	1559	780	-50.0%	1791	14.9%	1252	-19.7%
Ecosystems	species.yr	7.54	3.77	-50.0%	8.88	17.8%	5.76	-23.6%
Resources	M$	56.56	28.28	-50.0%	56.45	-0.2%	56.69	0.2%
**Total**	MPt	84.20	42.10	-50.0%	91.69	8.9%	74.28	-11.8%

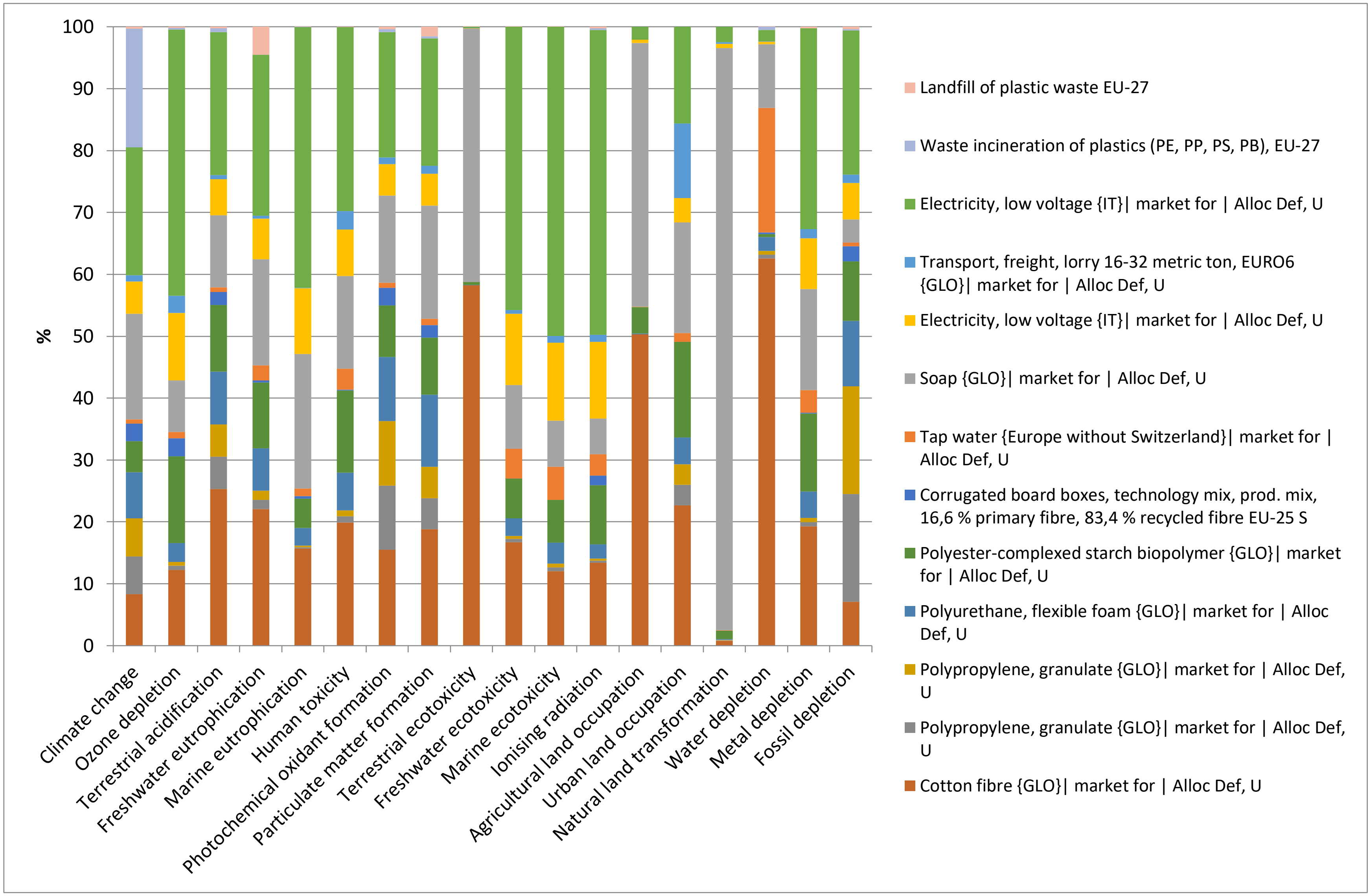

Figure S1. Impact assessment results for reusable face masks, basic use scenario (ReCiPe (H) Midpoint process contribution scores)

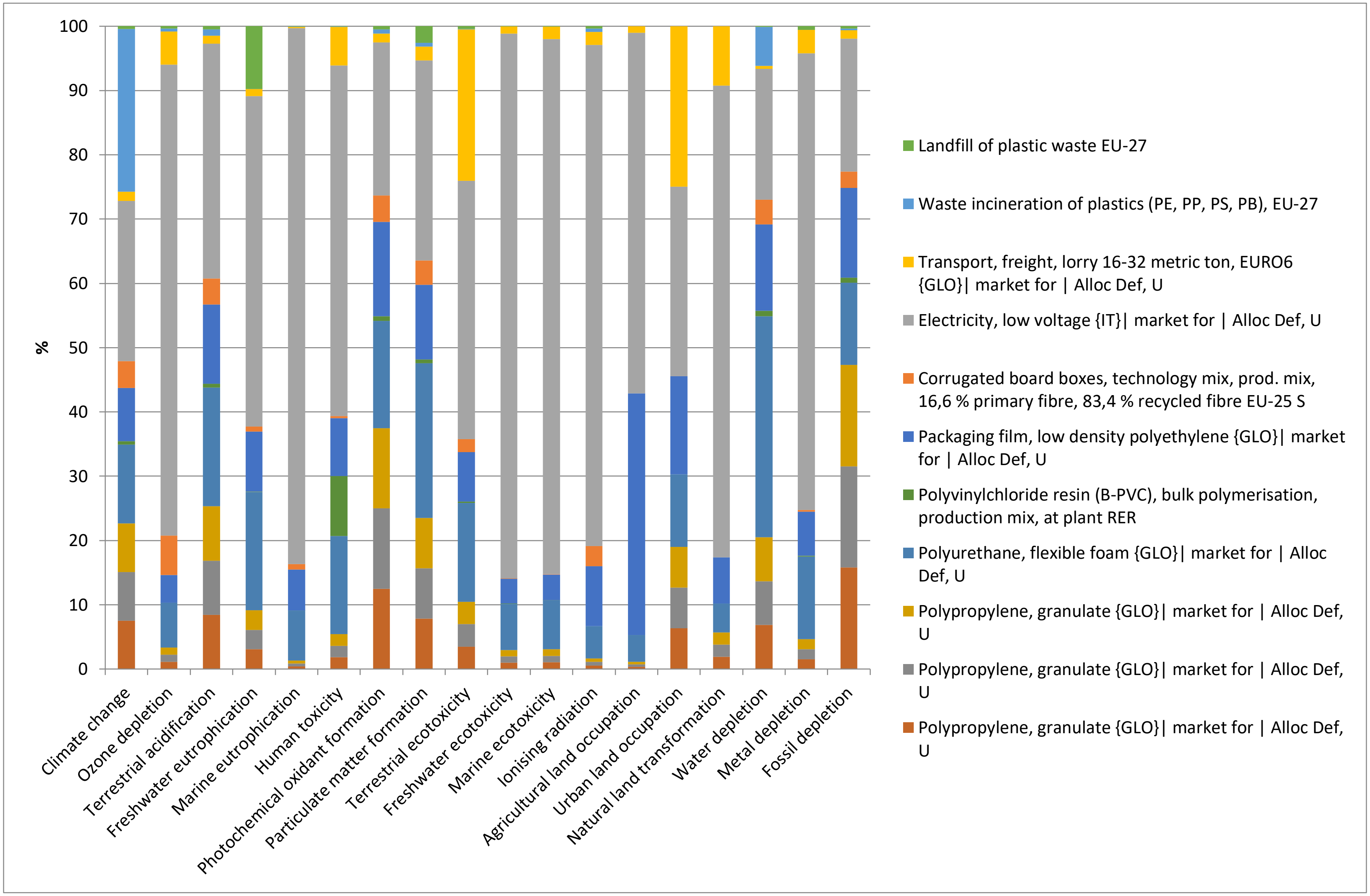

Figure S2. Impact assessment results for single-use face masks, basic use scenario (ReCiPe (H) Midpoint process contribution scores)
